# Poloxamer 407-Based Thermosensitive Emulgel as a Novel Formulation Providing a Controlled Release of Oil-Soluble Pharmaceuticals—Ibuprofen Case Study

**DOI:** 10.3390/ma14237266

**Published:** 2021-11-27

**Authors:** Kamil P. Grela, Dominik M. Marciniak, Bożena Karolewicz

**Affiliations:** Department of Drug Form Technology, Faculty of Pharmacy, Wroclaw Medical University, Borowska 211A, 50-556 Wrocław, Poland; dominik.marciniak@umw.edu.pl (D.M.M.); bozena.karolewicz@umw.edu.pl (B.K.)

**Keywords:** poloxamer 407, castor oil, in-situ emulgel, injectable emulgel

## Abstract

This article covers the design and evaluation of a novel drug vehicle: a thermosensitive, injectable, high-oil-content (50% *w*/*w*) emulgel providing a controlled release of lipophilic pharmaceuticals. Different vegetable (castor, canola, olive, peanut, grapeseed, linseed), mineral (paraffin) and semisynthetic (isopropyl myristate, oleic acid) oils were screened for ibuprofen (IBU) solubility and for their capacity for high-shear emulsification in a 17% (*w*/*w*) aqueous solution of poloxamer 407. Chosen emulgels were subject to a rheological evaluation, a syringeability test (TA.XT texture analyser; 2 mL syringe; 18 G, 20 G and 22 G needles) and a drug release study (48 h; cellulose membrane; 0.05 mol/L phosphate buffer at pH 7.4). Castor oil turned out to be an optimal component for IBU incorporation. Blank and drug-loaded castor oil emulgels were susceptible to administration via a syringe and needle, with the absolute injection force not exceeding 3 kg (29.4 N). The drug release test revealed dose-dependent, quasi-linear kinetics, with up to 44 h of controlled, steady, linear release. The results indicate the significant potential of high-oil-content, oil-in-water thermosensitive emulgel formulations as vehicles for the controlled release of lipophilic APIs.

## 1. Introduction

The use of aqueous solutions of poloxamer 407 (here abbreviated to P407; a common trade name is Pluronic^®^ F-127) as a thermosensitive medium providing a prolonged release of different active pharmaceutical ingredients (APIs) has been already thoroughly documented and reviewed in recent years [1,2,3]. Pharmaceutical researchers have studied compositions in which different APIs were simply dissolved in the poloxamer medium [4,5,6], encapsulated within micro- or nanospheres [7,8,9] or incorporated in macromolecular conjugates comprising P407 [10]. Binary compositions of poloxamer 407 and poloxamer 188 (thus Pluronics F-127 and F86, respectively) also appear to be a popular combination in poloxamer-based gels, where the addition of poloxamer 188 is usually used as a way to adjust the gelation temperature [9,11,12]. Additionally, mucoadhesive agents like hyaluronates or cellulose derivatives, which are predominantly compatible with poloxamers, are introduced to them to increase the mucoadhesive force and thus prolong the residence time within the application site [13,14,15,16].

While P407 is well-known for its solubilisation properties towards more or less lipophilic drugs—often bringing excellent improvements in the amount of API incorporated in drug form—these capabilities have their limits. Multiple attempts to overcome these limits have already been made, among which a technique of grafting other, more lipophilic molecules to the loose ends of the poloxamer’s polyoxyethylene chains seems to be a popular approach [17]. However, while this often happens to be an effective solution, it is always accompanied by the introduction of a new xenobiotic of unknown metabolism, toxicity and potentially unpredictable biological activities, which all have to be studied thoroughly prior to any further formulatory works [17].

With regard to these concerns, a more straightforward concept arose, which was to emulsify a significant amount of a well-known lipophilic excipient in an aqueous solution of pure P407. The main idea behind this concept was to create a reservoir for large quantities of lipophilic drugs within a thermosensitive poloxamer solution, but without the drawback of introducing new synthetics.

A literature review revealed some findings regarding poloxamer 407-based pharmaceutical emulsions. However, they mainly covered the use of P407 as a secondary excipient [18,19] or when used amongst many other ingredients in micro- or nanoemulsions [20,21,22,23,24], with only rare examples when the thermo–gelation phenomenon was addressed [25,26,27,28,29], although still with the presence of other emulsifiers.

Some of the most interesting examples of emulsions containing P407 are, to this date: (1) the employment of P407 as a secondary emulsifier in multiple, *w*/*o*/*w* emulsion, with Lactobacilli encapsulated in the inner aqueous phase [19]; (2) an intriguing composition called polyol-in-oil-in-water, with the inner hydrophilic phase composed of glycerol, 1,2-propanediol or 1,3-butanediol [30]; and (3) emulsification of up to three immiscible oils at once in a “Cerberus” emulsion by Ge et al. [31], where a 0.5% solution of P407 was capable of emulsifying silicone oil, fluorocarbon oil and a synthetic ester, ethoxylated trimethylolpropane triacrylate, forming non-spherical droplets.

Only three individual cases were found in which the P407 served both as an emulsifier and gelling agent. The first one was described by Täuber and Müller-Goymann [32], where a complex mixture of P407 with medium-chain triglycerides, propylene glycol, isopropyl alcohol and water was proposed as a carrier of ciclopirox olamine, an antifungal agent. However, the interesting situation described there was much more complex than obtaining a “thermosensitive emulsion”, because the tested formulations were actually described as translucent and isotropic; thus, they should not be considered macro- or miniemulsions, but rather microemulsions [33]. Macroemulsion was obtained in a more recent study, where sertaconazole nitrate was introduced into similar media, although these formulations did not form a gel when heated [34]. The second example of emulsification and gelation was described in two recent papers by de Souza Ferreira et al. [35,36], comprising a thorough, detailed approach to the development of a classical emulsion, in which poloxamer 407 served as a primary excipient being responsible for both emulsification and gelation properties. However, the proposed oil phase content was 0.25 to 0.75%, making it a quite different kind of formulation from the one presented in this paper. The third and most interesting case was published recently by Campanholi et al. [37]. Here, poloxamer 407 was applied in concentrations within 18% to 22% as an emulsifier and thermogelling agent in compositions with 0.2–0.3% of Carbopol and 15–25% of Copaiba oleoresin used as an oily active substance.

The present study was designed to formulate and evaluate a novel drug form of particular qualities: (1) being an oil-in-water emulsion with relatively high oil content; (2) formulated upon an already known and applied lipophilic media, providing a significant solubility of a model lipophilic drug; (3) dispersed in a thermosensitive aqueous solution of poloxamer 407; and (4) providing a prolonged release of a model lipophilic drug.

Whereas many oily excipients should be easily emulsified in poloxamer 407 solutions, some may bring suboptimal qualities like pharmaceutical incompatibilities, poor drug solubility or significant technological difficulties. Hence, the first step was to screen a group of oily media for their compatibility with poloxamer 407 solutions. Representatives of natural, semi-synthetic and mineral oils were selected out of the most popular oily components used in pharmaceutical formulations. Among natural oils, examples of non-irritant oils of different fatty acid profiles were selected in the hope to provide different solubilisation and emulsification capabilities. Castor oil was chosen as an interesting comparator due to its significantly different chemical structure (i.e., the presence of 12-OH groups). Free fatty acids were considered due to their lower molecular weight and lower viscosity when compared with acylglycerols, both qualities promising a different behaviour, especially by means of the solubility profile of the active substances [38]. However, this group was limited to oleic acid as saturated free fatty acids could not be used due to their unfavourably high melting points. Isopropyl myristate was included due to its widespread use as an oily ingredient in semisolid vehicles and as a potent solvent for many APIs. The choice of olive oil, oleic acid and isopropyl myristate was also justified by their potential use as penetration enhancers [38,39]. Less popular, “exotic” oils were intentionally avoided to not over-complicate the study.

Secondly, the solubility of ibuprofen, a model BCS class II drug, was to be assessed within the studied media to identify poor solvents and exclude them from further tests. Afterwards, a comprehensive rheological study, covering both blank and drug-loaded emulsions, was scheduled to assess the usefulness of obtained media and to provide a better understanding of the drug release kinetics, which were to be studied in a final step.

To the authors’ best knowledge, as of October 2021, there is no published research covering high-oil-content thermosensitive emulgels based solely on aqueous solutions of poloxamer 407 and dispersed oil phase. Thus, we resolved to call the very subject of this paper a novel formulation.

## 2. Materials and Methods

### 2.1. Chemicals

Poloxamer 407 was manufactured by BASF (Ludwigshafen, Germany) under the trade name Pluronic^®^ F-127 and sold by Sigma-Aldrich as a “BioReagent, suitable for cell culture” grade, containing 100 ppm butylated hydroxytoluene by declaration; we used it without further purification. Isopropyl myristate (98%) and olive oil (pharmaceutical grade) were supplied by Sigma-Aldrich (St. Louis, MO, USA). Oleic acid (pure) came from Chempur (Piekary Śląskie, Poland). Grapeseed oil (refined, food grade) was supplied by JCCoimbra Distribuição (Setúbal, Portugal). Castor oil (virgin), linseed oil (virgin), canola oil (refined) and paraffin—all of pharmaceutical grade—were supplied by Fagron (Kraków, Poland). Ibuprofen (pharmaceutical grade) was a kind gift from PPF Hasco-LEK, (Wrocław, Poland). Purified water meeting the pharmacopoeial standards was obtained in-house through ionic exchange and reversed osmosis. A buffer solution was made using analytical grade substrates supplied by ChemPur (Piekary Śląskie, Poland).

### 2.2. Stock Solutions Preparation

A 17% (*w*/*w*) poloxamer 407 solution (the given solution abbreviated here to P17) was prepared using the “cold” method [40,41], i.e., by sprinkling the polymer on the surface of cooled, pre-weighted water, closing tightly and placing it in a refrigerator. A clear solution was obtained overnight and was later stirred slightly to assure its homogeneity. Ibuprofen (IBU) solutions in castor oil (ORC) at the concentrations of 5%, 10% and 15% IBU (*w*/*w*) were prepared using a magnetic stirrer, with a moderate heating up to 40 °C.

### 2.3. Sample Preparation

**Figure 1:**
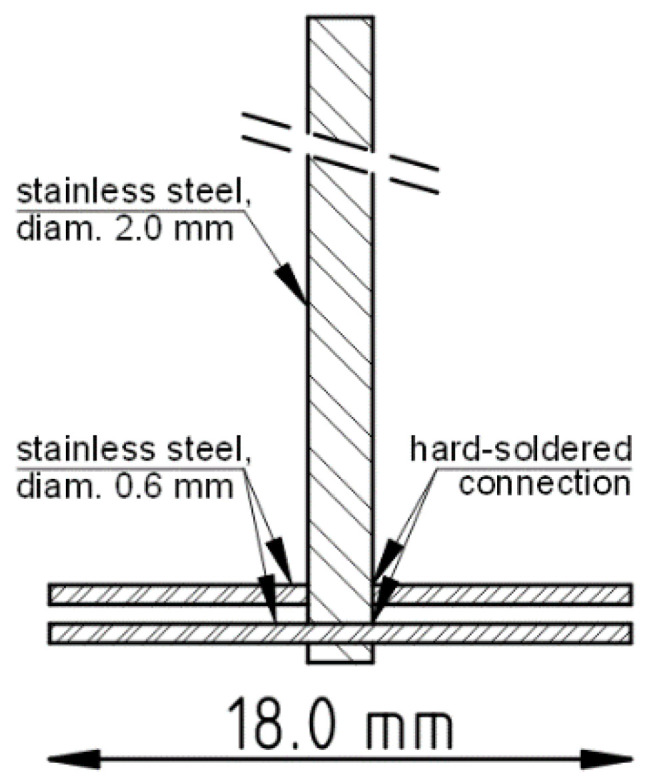
Technical sketch of the stirrer used in low-volume homogenisation of emulgels.

Every studied emulsion was prepared in a standardised manner in a uniform glass vial (volume: 15 mL, inner diameter: 25 mm). First, the aqueous phase was pre-weighted into a vial, then the oil phase was added, and the contents were immediately stirred with a high-shear mixer using a custom-made stirrer, as shown in Figure 1. The mixing time was 120 s, and the rotational speed was 8000 min^−1^ (RPM). After emulsification, all samples were stored in a laboratory refrigerator (5 °C) in airtight containers protected from natural or artificial light. IBU-containing emulsions were prepared in the same way, i.e., through the emulsification of a pre-made ORC–IBU solution in P17.

### 2.4. Emulsification Capacity

At first, an initial amount (5 g) of P17 solution was weighed into a vial, then a portion of the chosen oil, corresponding to 5% of the total mass of a mixture (by weight), was added upon its surface. This composition was stirred for 120 s in the setup explained earlier, and then the resulting mixture was evaluated in terms of colour, consistency and flow. If a given mixture showed qualities of an emulsion (i.e., white or off-white colour, uniform and smooth consistency), it was left undisturbed for 5 min to observe any possible signs of destabilisation. If no rapid breakdown was noticed, the next portion of oil (5% of the total mass of the mixture) was added, and the mixture was stirred and evaluated again. This process was repeated for every oil, in the same 5% increments, up to the moment when a given mixture showed first visible signs of a breakdown or when any emulsification difficulties occurred. If no breakdown was observed, yet the total emulsification seemed impossible, the mixture was cooled in a refrigerator (to ease the emulsification by decreasing the viscosity of the aqueous phase) and mixed again, oftentimes successively. When an apparent emulsification limit (i.e., rapid emulsion breakdown or total emulsification inability) was approached, two consecutive samples were prepared in the same way to confirm the result. The maximal oil content providing a stable emulsion was recognised as the emulsification capacity. In cases of unrepeatable results, the lowest value from three repetitions was chosen.

Afterwards, a set of fresh samples of emulsions at their “boundary” compositions, as listed under “Emulsification capacity” in Table 1, were stored in a refrigerator for 3 months for further observation for any obvious stability issues.

The structure of obtained emulsions was evaluated by a dissolution method, in which minute aliquots of emulsions were transferred to distilled water, in which their behaviour was observed. A quick dispersal was considered as an indication of oil-in-water structure, while a floating undispersed droplet as of water-in-oil structure. These results were later confirmed by the staining method: a diluted solution of hydrophilic dye (methylene blue) was mixed thoroughly with tested emulsions, and the results were examined under a microscope.

### 2.5. Rheometry

Rheological properties of studied formulations were evaluated by rotational rheometry, using a Brookfield RVDV-III+ rheometer (AMETEK Brookfield, Middleboro, MA, USA; formerly: Brookfield Engineering Laboratories) in a cone-plate, controlled shear rate setup. The rheometer’s head was originally equipped with a water jacket, and the temperature control was provided by a circulating water bath with declared stability of ±0.005 °C. The types of cones (CP40/CP51) and applied shear rate ranges were selected with proper consideration of apparent viscosities and their relation to the measurement range of the apparatus; hence, they were different for free-flowing formulations than for gelated formulations.

The viscosities were measured at three different temperatures: 20 °C, 25 °C and 37 °C, in each case over a decade of shear rate, with necessary pre-shearing period and stabilisation of the reading before the collection of each consecutive data point. In addition, in measurements taken at the gel state (in every case at 37 °C and in some cases at 25 °C), a prolonged pre-shearing period (180 s) was implemented to ensure a uniform sample distribution before any measurement.

**Figure 2:**
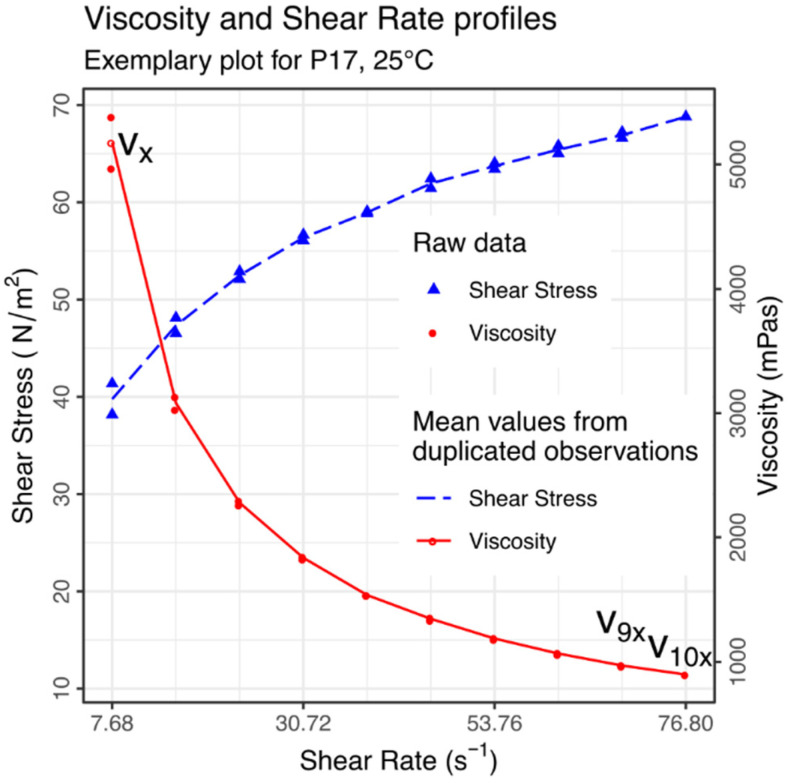
An exemplary result of a rheological measurement performed at 25 °C. Both the Shear Stress and Viscosity are plotted against the Shear Rate. Duplicated values in the range *v_x_*:*v*_9_*_x_* (here 7.68:69.12) were averaged before any further calculations of the Pseudoplasticity Index. The *v_x_* and *v*_10_*_x_* labels indicate the viscosities used to calculate the Pseudoplasticity Index from individual measurements.

A parameter called Pseudoplasticity Index (PI) [42,43] was extracted from the results of the above rheological tests. PI was calculated as:

(1) $$\mathrm{PI}=\frac{v_{x}}{v_{10x}},$$

thus as a ratio of initial ($v_{x}$) to terminal ($v_{10x}$) viscosity of a sample, when measured over a decade of Shear Rate (see Figure 2 for visual explanation). This parameter was considered a measure of the shear-thinning phenomenon; therefore, of the apparent pseudoplasticity of a given sample.

**Figure 3:**
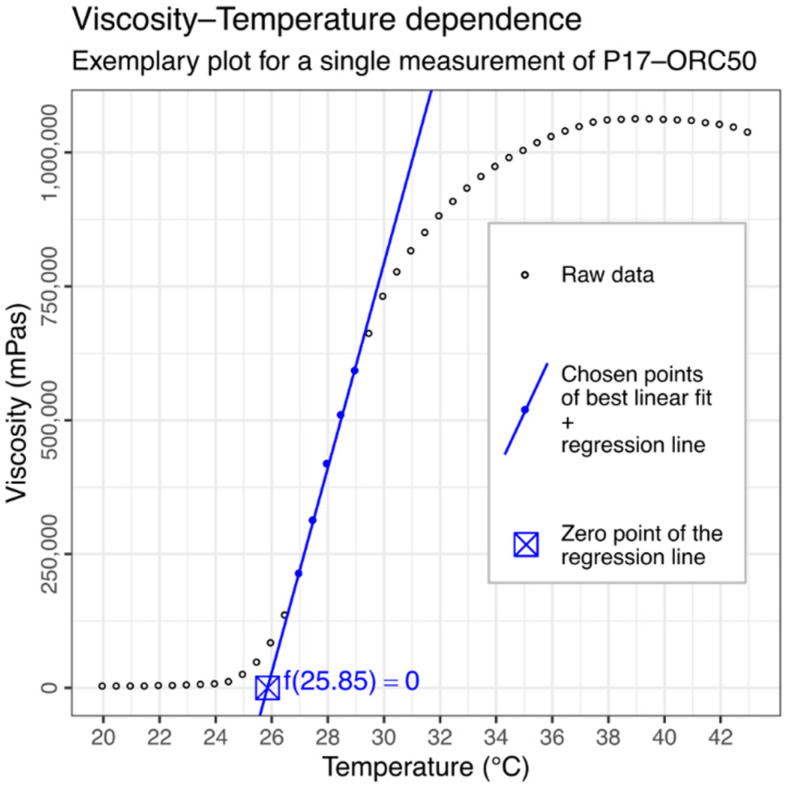
An exemplary result of a ‘viscosity vs. temperature’ test. Five points, shown as blue dots, were algorithmically chosen as the most linear part in the region of rapid viscosity growth. Tgel was then calculated as a zero point of the regression line.

A viscosity vs. temperature relation was evaluated through another rheological measurement, in which the viscosity was recorded at given temperature intervals (every 0.5 °C) during a constant, continuous shearing (SR = 0.192 s^−1^), with linear increase (1.0 °C/min) and then decrease (0.5 °C/min) in temperature. These viscosity profiles were used to determine the gelation temperature (Tgel). The Tgel parameter was calculated through a linear regression upon an algorithmically selected subset of five data points constituting the most linear section of the steep, ascending part of the viscosity vs. temperature profile (see Figure 3). The zero point of each regression line was considered as the gelation temperature (Tgel).

Every kind of rheological measurement described above was performed in triplicate.

In order to correctly compare the viscosities of different formulations, two common Shear Rate values were chosen, which were precisely 38.4 s^−1^ in the liquid state (thus at 20 °C and 25 °C for most cases) and 0.384 s^−1^ at the gel state (thus at 37 °C for every formulation and also at 25 °C in case of the IBU-loaded emulsions).

### 2.6. Tube Inversion Method (TIM)

TIM test, as described earlier [44,45,46], was performed to visually confirm the gelation phenomenon. A 5.0 mL aliquot of each formulation was transferred to a clear glass vial and sealed tightly. The vial was submerged in a water bath, which temperature was changed gradually in the range of 20–40 °C in 0.5 °C steps. After every temperature increment, the vial was left undisturbed for 10 min, and then, while still being submerged, it was carefully turned upside-down and left in this position for 2 more minutes. The mobility of the content was then evaluated by two investigators, using a three-point scale: (1) liquid—freely flowing at the moment of reversal; (2) soft gel—slowly flowing during the 2 min stage; and (3) hard gel—not deforming during the 2 min stage. Afterwards, the vial was returned to its original position, the temperature setting was increased, and the successive steps were repeated. The temperature at which a hard gel was observed for the first time was considered a gelation temperature (TIM-Tgel). The 10-min interval was chosen concerning the time needed for the thermal equilibration of the bath and the sample, which was earlier determined to be 7:30 ± 0:20 min in an identical setup (bath, water level, vial type, sample amount).

### 2.7. Syringeability

This test, intended to evaluate the possibility of emulsion administration via injection [47], was carried out using a TA.XT Plus texture analyser (Stable Micro Systems, Godalming, UK) in a setup similar to the one described by Burckbuchler et al. [48]. The apparatus was equipped with a syringe rig allowing to measure the force needed to move the syringe’s plunger at a given linear velocity. One type of syringe (2 mL, Braun Injekt; B. Braun Melsungen AG, Melsungen, Germany) and three different needle gauges (22 G × 30 mm, 20 G × 40 mm and 18 G × 40 mm; UNIT Medical (Intergos, Bielsko-Biała, Poland); see Table S1 in Supplementary Materials for more details) were applied to provide a broad spectrum of conditions. The surface area of the plunger was determined to be 0.7595 cm^2^ and, as such, was used to calculate the linear plunger velocities needed to provide a desired output flow rate at levels: 0.25, 0.5, 1.0, 2.0, 4.0 and 8.0 mL/min. Syringes were filled up to 3.0 mL and, usually, one syringe was utilised to perform three separate measurements at different flow rates, each over a 10 mm movement range. The force was recorded in 2-millisecond intervals during the continuous extrusion phase. Data from the first 2 mm and last 1 mm of each cycle was excluded, and the remaining part was averaged to obtain a singular value from every measurement. Measurements were performed in triplicate for each needle-flow rate combination. Formulations were extruded into air at ambient temperature (25 ± 1 °C). The empty syringe’s resistance was measured after a formulation was tested to account for the specific lubricating properties of a given formulation. This force was later averaged for a given formulation and flow rate and subtracted from the raw (gross) extrusion force to obtain the net force.

### 2.8. IBU Determination via HPLC

Apparatus: Agilent 1260 Infinity, Agilent Technologies Inc. (Santa Clara, CA, USA). Column: ODS Hypersil 5 µm, 150 mm × 4.6 mm, Hewlett-Packard (Waldbronn, Germany).

Basic method used for determination of IBU in drug release test: Phase A: 0.1% TFA (trifluoroacetic acid) in purified water, Phase B: Acetonitrile. Elution: 55% B, 1.2 mL/min; Rt 3.55 min. Detection: UV-DAD, 220 nm. Calibration curve: c [mg/mL] = PeakArea 2.4935 × 10^−5^ − 0.00098; R = 0.99987, *n* = 18.

A modification with an oil-compatible mobile phase was applied in the determination of IBU solubility in lipophilic media: Phase A: 0.1% TFA in purified water, Phase B: Isopropyl Alcohol. Elution: 40% B, 1.2 mL/min; Rt 5.20 min. Detection: UV-DAD, 264 nm. Calibration curve: c [mg/mL] = PeakArea × 8.3243 × 10^−4^ + 0.0008; R = 0.99974, *n* = 18.

### 2.9. IBU Solubility in Chosen Vehicles

An anticipated excess of IBU was added to 5.0 g aliquot of each solvent, and then every mixture was stirred in a closed container for 72 h at room temperature (22 ± 1 °C). The resulting saturated solutions were left to settle for 24 h, then a 1 mL aliquot of each was withdrawn and centrifuged for 10 min at RCF = 10,080. Samples were diluted in an aqueous–organic solvent mixture, before the IBU content was determined via HPLC.

### 2.10. Drug Release Test

Apparatus: Hanson SR8+ dissolution apparatus + “Model B” immersion cells [49]. Dialysis membrane: Spectra/Por 6, MWCO: 1000 Da, regenerated cellulose, wall thickness 60–65 µm; Repligen Corporation (Waltham, MA, USA; formerly: Spectrum Labs).

A drug release test was performed in accordance with USP chapter 1724, in a modified paddle apparatus equipped with 150 mL flat-bottom vessels and small-volume “Model B” immersion cells [50]. The drug reservoir volume was 0.53 cm^3^, and the membrane surface area was 1.77 cm^2^ [49]. A dilute (0.05 mol/L) phosphate buffer of pH 7.4 ± 0.05 was used as a receptor medium to simulate the pH of the human extracellular fluid and at the same time provide sufficient IBU solubility (4.64 ± 0.08 mg/mL) to fulfil the criteria of sink conditions [51,52]. The test was carried out at 37 ± 0.5 °C with a stirring speed of 50 RPM. Receptor medium aliquots were auto-sampled in unequal time intervals covering the whole 48 h test period. The final pH of the receptor medium was, in each case, confirmed to be within ±0.05 from the initial value. The receptor medium remained clear during the whole test, i.e., no leak was observed in any cell, and no precipitation of any solute was observed.

### 2.11. Statistics and Data Presentation

Data processing, further statistical analyses and data visualisations were performed in the R environment (v3.6.3) using RStudio v1.4. Results are presented as mean values ± standard deviation, calculated from three measurements unless otherwise specified. Box plots show mean values (horizontal lines), ± standard errors (boxes) and ±95% confidence intervals (whiskers). One-way ANOVA was applied to evaluate the statistical significance of chosen differences. ANOVA was preceded by the evaluation of critical conditions (Shapiro–Wilk normality test, Levene’s test for variance uniformity), and the post-hoc *p*-values were generated by Holm’s method. Every linear and non-linear regression was calculated using a classical “least squares” loss function, although the final results of these regressions were in some cases evaluated and compared by an adjusted sum of squares, described later in more detail.

## 3. Results and Discussion

### 3.1. Emulsification Capacity/IBU Solubility

**Figure 4:**
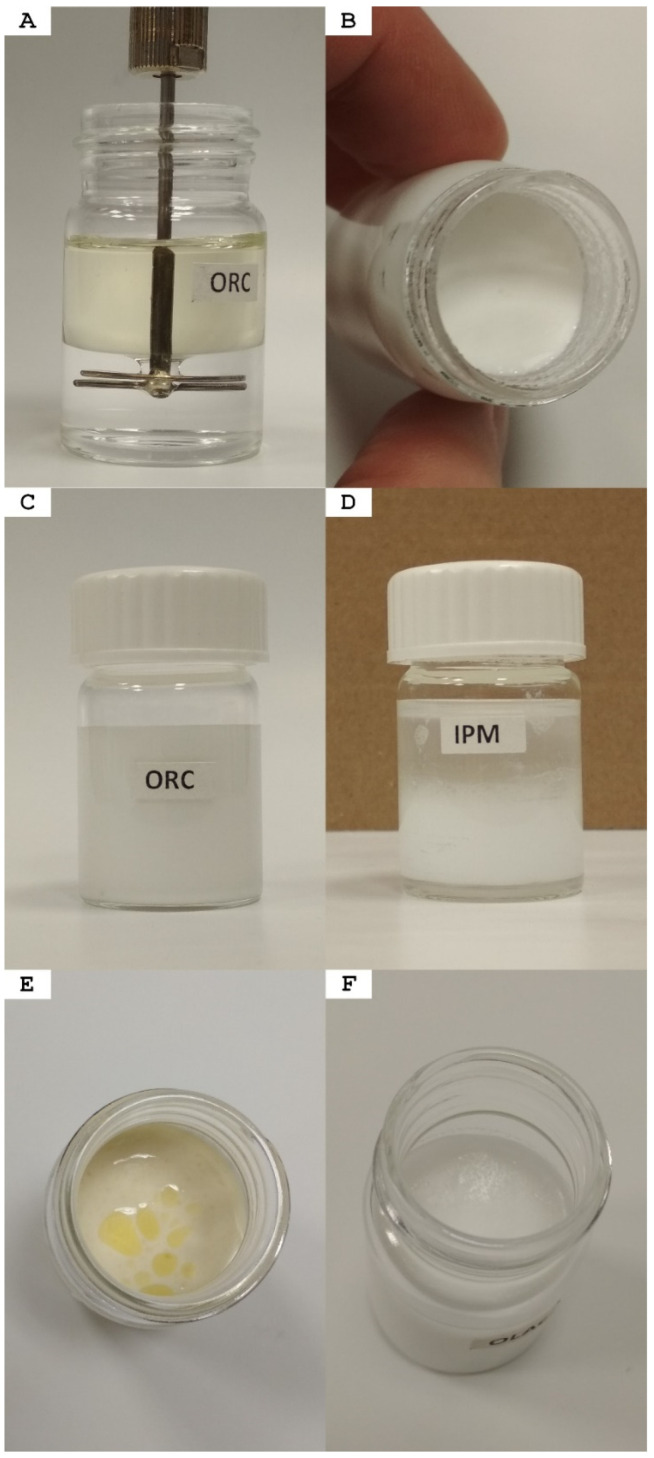
A brief look at the homogenisation process: (**A**) homogenisation setup; (**B**) usual appearance of a freshly prepared emulsion; (**C**) P17–ORC50 blank emulsion homogenised and transferred to a new vial; (**D**) failed homogenisation attempt in P17:IPM at 1:1 composition; (**E**) partial phase separation in P17–OLN50 emulsion observed after 1 month; (**F**) P17–OLAc50 emulsion (*w*/*o*) solidified in a refrigerator (4 °C), moderate signs of phase separation were visible when thawed.

The emulsification study revealed that each vehicle was readily emulsifiable in the P17 solution. Moreover, in most cases, a 50% oil emulsion could be obtained simply by mixing equal amounts of oil and P17 (Figure 4B,C). The IPM turned out to be the only exception in which a 1:1 proportion was never successfully emulsified (Figure 4D). Thus, it seemed that an initial composition of 10% of IPM was a good starting point to obtain a stable *o*/*w* emulsion, which later could be easily enriched with oil up to 65%.

A usual result of emulsification was a creamy, free-flowing emulsion, with a silky and slippery touch, quickly increasing its viscosity on contact with a warm hand. The final colours were affected by the original colour of oil: thus, they varied from snow-white for PAR, IPM and OLAc; through white or off-white for OAR, ORC and ORP; light yellowy-greenish for OOL and OGR; and, finally, light pastel yellow for OLN. For photographs of emulsions, please refer to Figures S5–S7 in Supplementary Materials.

The overall emulsification capacity results suggested that a 50% oil phase content should be a safe and convenient composition for every kind of oil, and so it was applied for any further studies, whether or not they involved IBU addition.

In the matter of IBU solubility (see Table 1; Figure S1 in Supplementary Materials), the tested media formed three visible groups: (1) paraffin alone, being a relatively poor solvent; (2) conventional vegetable oils, providing nearly uniform solubility at around 56 mg/g; and finally a group (3) consisting of a less-conventional media, which were capable of dissolving far more than 100 mg/g of IBU.

**Table 1:** Summary of media properties.

Medium Abbreviation and Full Name		Emulsification Capacity in P17 Solution (Oil Phase Content, by Weight)	Storage Stability over 3 Months	Emulsion Structure in Studied Concentrations	IBU Solubility ± sd (mg/g)	Density (g/mL)
OAR	Peanut Oil	65%	+	*o*/*w*	56.6 ± 1.9	0.915 *
OGR	Grapeseed Oil	65%	+	*o*/*w*	52.3 ± 0.4	0.923 *
OLN	Linseed Oil	60%	slow breakdown after 2+ weeks (Figure 4E)	*o*/*w*	60.3 ± 0.8	0.931 *
OOL	Olive Oil	65%	+	*o*/*w*	55.1 ± 0.7	0.913 *
ORC	Castor Oil	>85%	+	*o*/*w* up to 85% oil, phase inversion into *w*/*o* at 90%	160 ± 3.6	0.958 *
ORP	Canola (rapeseed) Oil	65%	+	*o*/*w*	56.9 ± 0.3	0.917 *
PAR	Paraffin	80%	+	*o*/*w*	13.6 ± 0.4	0.827–0.980 *, 0.861 ^$^
IPM	Isopropyl Myristate	>75%	+	*o*/*w*, gradual inversion at higher %	125.9 ± 0.7	0.853 *
OLAc	Oleic Acid	>75%	solidifies if refrigerated	*o*/*w* up to 20% oil, phase inversion into *w*/*o* at 25%	171.8 ± 0.8	0.892 *

* literature data, ^$^ measured value.

While natural oil derivatives such as oleic acid and isopropyl myristate showed high IBU solubility, both brought some technological disadvantages. Oleic acid formed only a water-in-oil emulsion when used in higher concentrations, which generally put it outside of the scope of this study; furthermore, its relatively high melting point (16.3 °C) would also question the stability of dispersions in case of low-temperature storage (Figure 4F). Isopropyl myristate showed some difficulties in the technological process, i.e., any composition richer than 15% of IPM had to be prepared through at least a two-step process. Hence, castor oil turned out to be a promising choice for a further drug-involving study due to its excellent IBU solubility and convenient technological behaviour. When compared to other lipophilic media, its relatively high density was also a good theoretical predictor of emulsion stability [53] due to a relatively low difference in phase densities and thus lower sedimentation/creaming forces.

IBU-loaded emulsions were prepared by mixing the P17 solution with equal amounts of 5-, 10- or 15% solutions of IBU in ORC (details shown in Table 2). They were obtained as easily as blank P17–ORC50 and were visually no different from it (Figure S7 in Supplementary Materials).

### 3.2. Rheology and Gelation

**Figure 5:**
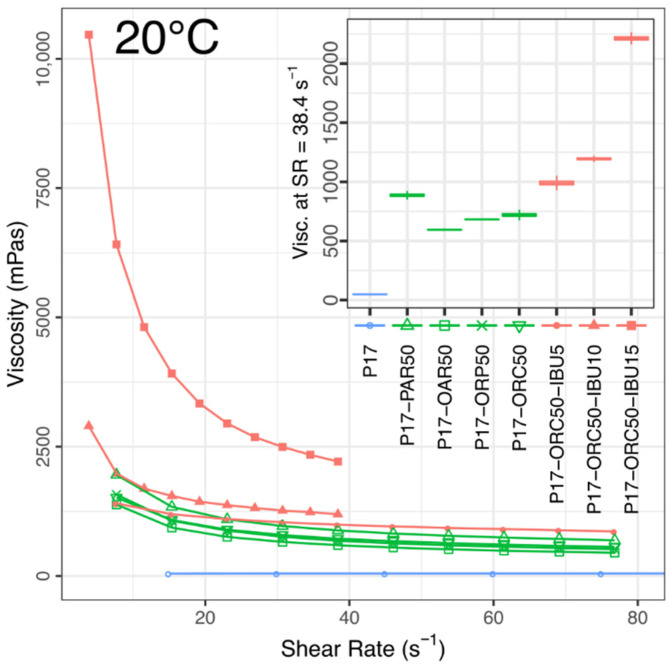
Viscosities vs. shear rate in blank and drug-loaded emulsions compared to the P17 solution. 20 °C, mean values, *n* = 3. Inset shows mean viscosities at SR = 38.4 s^−1^ (±std error, ±95% conf. interval). Different shear rate ranges were dictated by the rheometer’s capabilities.

**Figure 6:**
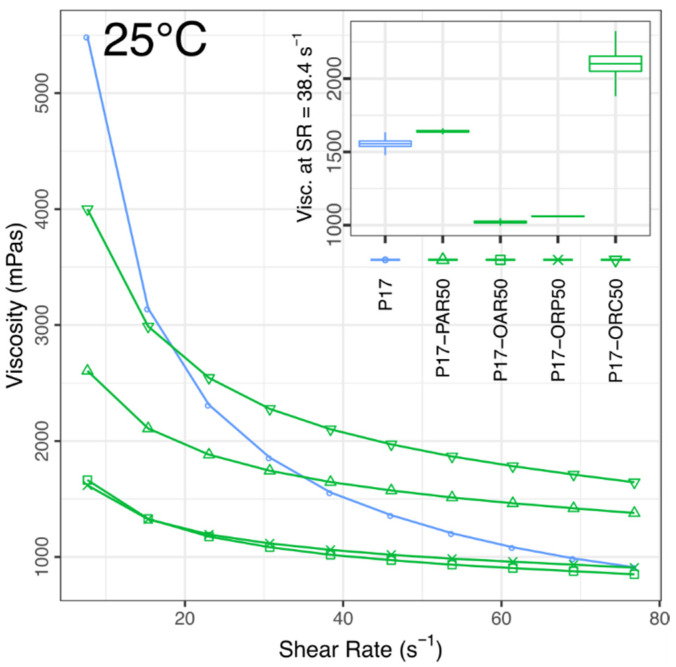
Viscosities vs. shear rate in blank emulsions compared to the P17 solution. 25 °C, mean values, *n* = 3. Inset shows mean viscosities at SR = 38.4 s^−1^ (±std error, ±95% conf. interval). At 25 °C all IBU-loaded formulations formed plastic gels, which had to be tested separately (see Figure 7).

**Figure 7:**
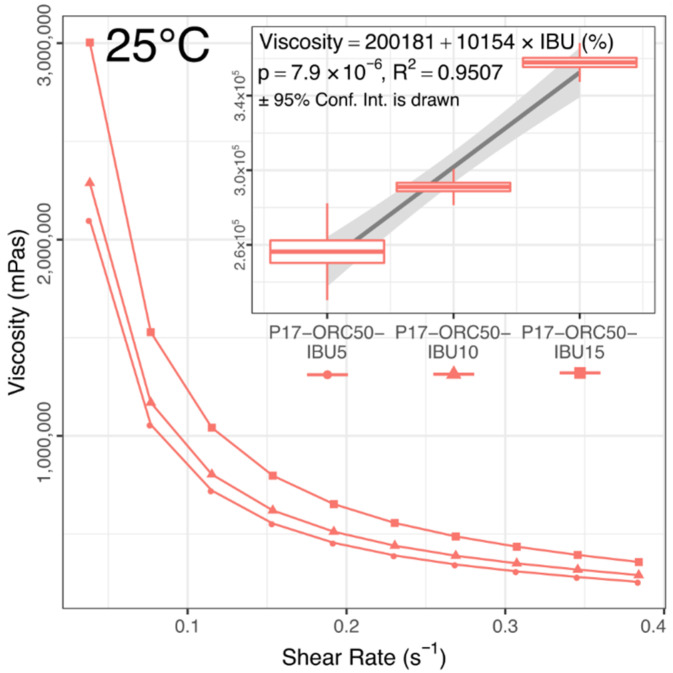
Viscosities vs. shear rate in IBU-loaded emulsions. 25 °C, mean values, *n* = 3. Inset shows mean viscosities at SR = 0.0384 s^−1^ (±std error, ±95% conf. interval) with a quasi-linear correlation between the viscosity and the IBU content. Blank formulations were still liquid at these conditions; thus, they were tested separately, see Figure 6.

**Figure 8:**
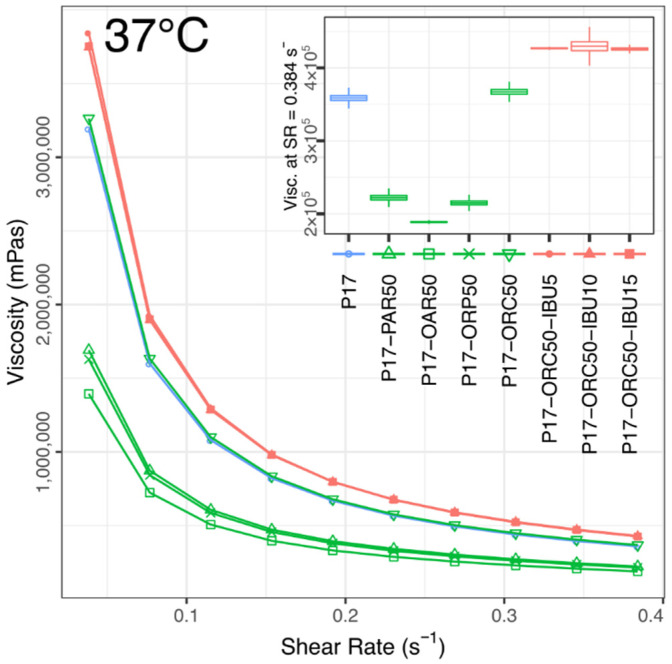
Viscosities vs. shear rate in all tested formulations. 37 °C, mean values, *n* = 3. Inset shows mean viscosities at SR = 0.0384 s^−1^ (±std error, ±95% conf. interval).

The rheological study revealed that every studied emulsion possessed undoubtedly non-Newtonian characteristics. Their common quality was a relatively high shear sensitivity, which caused the viscosity to drop considerably over the range of shear stress applied during the tests. The pseudoplastic behaviour, typical for poloxamer gels [54], was particularly noticeable in gelated emulsions but also occurred (yet to a smaller extent) in every emulsion tested in a liquid state (see Figure 5, Figure 6, Figure 7 and Figure 8).

At 20 °C, the viscosities of different blank emulsions varied significantly for up to 20% from their overall mean value. Still, all of them stayed in one general region of viscosity, which was 590–890 mPas (at SR = 38.4 s^−1^), as seen in the inset in Figure 5. The mean viscosity of all blank emulsions was approximately 15 times higher than that of pure P17 solution (48.8 mPas). Any addition of IBU caused a significant increase of viscosity, but no obvious gelation was observed even in the most concentrated emulsion (P17–ORC50–IBU15). A closer look at the viscosity curves in Figure 5 and Pseudoplasticity Indices (Table 3; Figure S2 in Supplementary Materials) reveals very similar pseudoplasticity levels (thus general rheological behaviour) among blank emulsions, and—again—a strong, positive and non-linear effect of IBU on this measure.

Measurements performed at the intermediate temperature (25 °C, Figure 6 and Figure 7) revealed a significant viscosity rise in all formulations compared to the earlier measurements at 20 °C. The mean viscosity of blank emulsions doubled (720 → 1456 mPas), while the individual values also became twice as diversified as earlier (now ±40% of the mean). All IBU-loaded emulsions formed plastic gels, forcing different measurement conditions, where the viscosities reached an average level of 300,000 mPas and showed a linear correlation with the IBU content (see Figure 7).

The viscosity of pure P17 increased over 30-fold, reaching the blank emulsions’ average level. P17 showed quite different rheological behaviour, which manifested through a Pseudoplasticity Index six times higher than before and significantly overtaking that of blank emulsions (Table 3; Figure S3 in Supplementary Materials).

The general viscosity growth with such an increase of temperature is the expected behaviour of poloxamer solutions [55], yet the specific relations observed here between P17 and blank emulsions need some more consideration to be fully understood. The gelation temperatures, presented in Table 3, seem to help in this matter. Namely, at 25.0 °C, the P17 approaches its Tgel, meaning the beginning of a rapid viscosity growth should be expected, with the corresponding formation of a so-called “soft gel”. On the other hand, the 5 °C rise from 20.0 °C to 25.0 °C, while bringing the P17 to the verge of its gelation onset (25.17 °C), seemed to play an obviously less significant role in the thermodynamics of PAR, OAR and ORP emulsions, all being at least 1.9 °C apart from their gelation temperatures. At the same time, the ORC emulsion, which had the lowest Tgel of all blank emulsions (25.83 °C), also slowly approached the gelation onset, and that may explain why the highest relative viscosity increment (719 → 2100 mPas) was observed in its case. However, this viscosity increment was not accompanied by any increase of PI, which suggests that any manifestation of a “soft gel” may still not form at this moment. Moreover, in both P17 and P17–ORC50, the proximity of Tgel appeared also to negatively affect the repeatability of the measurement, which is visible through the increased measurement errors presented in Figure 6, and which also appeared to confirm the above conclusion.

Finally, the mechanics behind the decreased PI in blank emulsions (a drop from 2.92 to 2.02, on average) may be explained in such a way that (1) over the aforementioned 5 °C step, the viscosity of the aqueous (dispersing) phase increased significantly, yet not enough to actually induce the formation of a pseudoplastic “soft gel” (see TIM-Tgel values in Table 2), while (2) at the same time, the viscosity of the oily (dispersed) phase dropped significantly (see Table 4 for ORC viscosities), which eased any plastic deformation of oil droplets under shear. Due to the decreased viscosity of the dispersed phase, the deformation of droplets consumed less energy, and the viscosity of the oil phase therefore played a lesser part in the total viscosity of the emulsion. Since it is known that the pseudoplastic behaviour in emulsions is caused mainly by the droplets of the dispersed phase undergoing a temporary deformation [56,57], this may explain why the pseudoplasticity of studied emulsions lowered with temperature increase despite the growth of the total viscosity, which was caused mainly by the dispersing phase.

At 37 °C, every formulation turned into a highly-pseudoplastic gel (PI 7.38–9.01, Figure S4). Their viscosities were relatively less diversified than at lower temperatures (inset in Figure 8; compared with Figure 5, Figure 6 and Figure 7), yet three general groups were still visible, where: (1) all IBU-loaded emulsions reached a uniform top level; (2) the P17 hydrogel and ORC emulsion took a common, slightly (15%) lower value; and (3) the remaining blank emulsions gathered at the lowest level, which was approximately a half of the viscosity of IBU emulsions.

**Figure 9:**
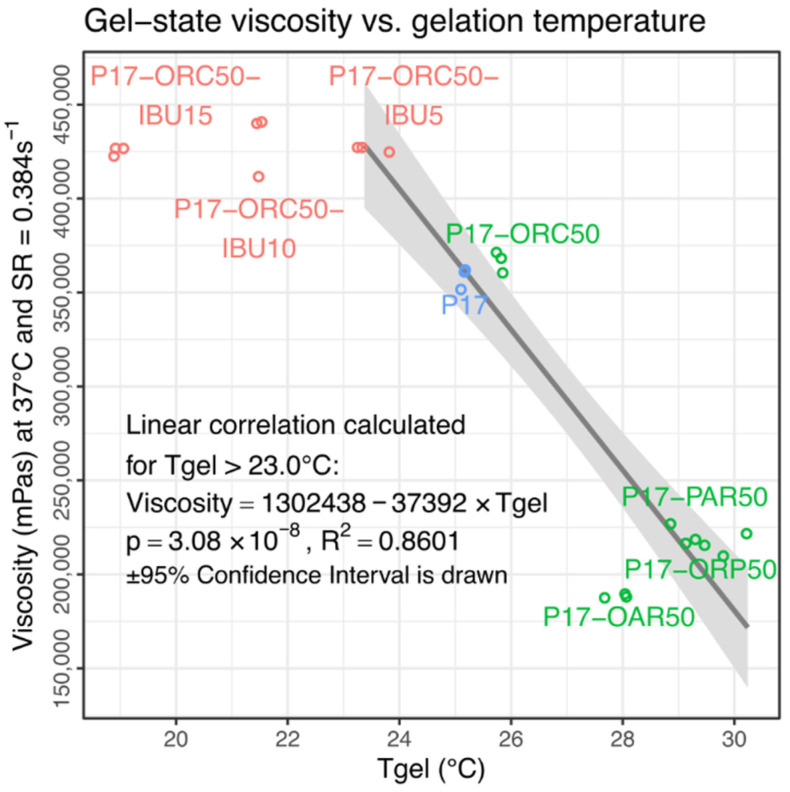
An overview of gel viscosities vs. corresponding Tgel values. A negative, linear correlation between viscosities measured at 37 °C and Tgel is observed in formulations less prone to gelation.

A general negative relationship was observed between these viscosities and corresponding gelation temperatures. After the exclusion of the two lowest Tgel levels, which appeared to be absolutely unrelated to the viscosity, for the six remaining emulsions (characterised by a Tgel of 23 °C or higher), the gel viscosity turned out to be a quasi-linear reflection of the gelation susceptibility expressed via Tgel (Figure 9).

The above viscosity–Tgel correlation suggests that at a high temperature (or in a “fully-gelated” state), the emulgel properties may result not from the sole presence of a dispersed phase but from the influence of a given oil or API, being exerted specifically on the gelation ability and manifested via Tgel shift. Moreover, the correlation shape and region suggest the occurrence of a critical Tgel value of about 23–24 °C, which should not be exceeded for the emulgels if their viscosity is intended to be fully developed at 37 °C.

Knowing the significant chemical differences between the castor oil, paraffin and remaining vegetable oils used in this study, the presence of three specific groups visible in Figure 9 may also be explained.

The first group is located in the bottom-right and comprises PAR/OAR/ORP emulsions, which shared similar viscosities, Tgel and PI values (Table 3; Figure S4). While their viscosities at lower temperatures (20 and 25 °C, Figure 5 and Figure 6) were not that equal, it seems that their effect on the gelation mechanism was quite similar and was based primarily on lipophilic interaction between “fat” hydrocarbon chains and poloxamer’s central, lipophilic, poly(propylene oxide) chain. The proximity of PAR emulsion to triglyceride emulsions suggests that the ester groups present in vegetable oils seem to not affect the gelation process in a significant way.

The second group of formulations is formed by P17 and blank P17–ORC50 emulsion, which shared a very similar Tgel value and almost identical viscosities and PI values (Figure 9 and Figure S4, Table 3). Interestingly, the effect of castor oil appeared to be nearly the same as of other oils only when measured at 20 °C. It tended to change in a unique manner with temperature increase, towards a somehow “invisible” influence of ORC on the cross-section of rheological properties measured at 37 °C. However, considering the chemical structure of ORC, this “invisible” influence should be rather understood as resultant of (1) a “fat” interaction as in other oils, lowering the viscosity and rising the Tgel; and (2) ORC’s individual influence, caused presumably by hydrophilic interactions between poloxamer and C12-OH groups of ricinoleoyl entities and being responsible for an opposite counter-effect, neutralising the gelation disturbances from “fat” chains.

**Figure 10:**
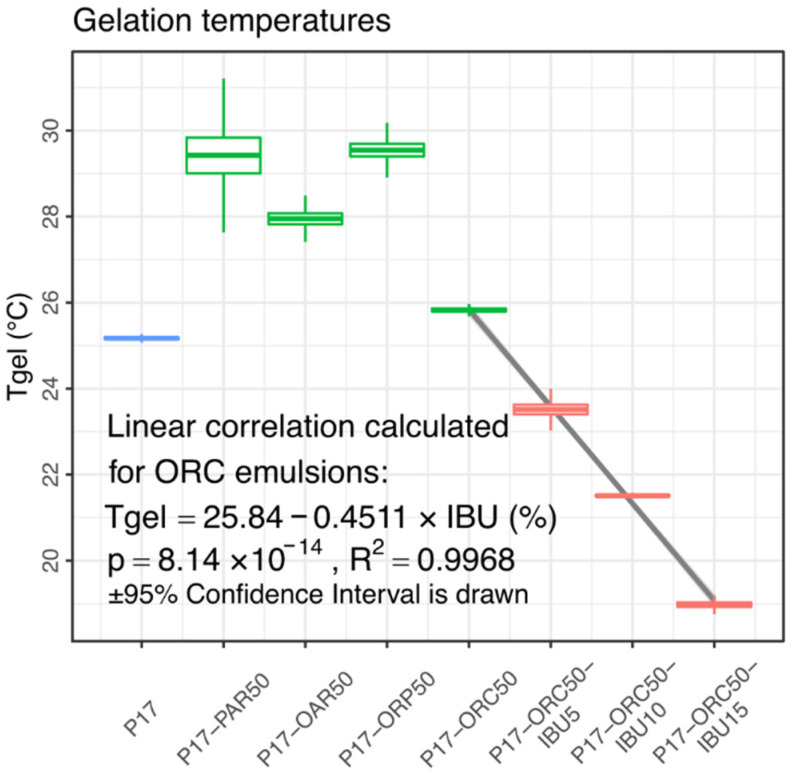
Gelation temperatures in tested formulations. A strong negative linear correlation between Tgel and IBU content is observed in the ORC series over the whole range of IBU content.

The third group, visible at the top in Figure 9, consists of IBU-loaded emulsions, which all overgrew the pure hydrogel and P17–ORC50 emulsion and reached a peak viscosity. This effect was caused by a general positive influence of IBU on the gelation process, and it was expected based on the authors’ previous experiments (not cited here) and literature findings [25]. While the top viscosities were affected in a binary way, regardless of the IBU content, an almost perfectly linear correlation between the IBU content and Tgel value was found (Figure 10). Notably, this impact could not be studied to this extent—i.e., up to several percent of IBU in a mixture—without the presence of oil phase because the solubility of IBU in pure P17 is significantly lower than concentrations applied in emulgels. An attempt to measure this solubility was unsuccessful due to the extremal influence of IBU on the viscosity and gelation of P17. However, the value was estimated as approx. 12 mg of IBU per 1 g of P17.

It should be noted that the specific influence of any API/excipient on the Tgel may be preliminarily estimated even in a composition with pure poloxamer, i.e., before any serious formulatory studies.

TIM study served as a more tactile approach to the gelation phenomenon and allowed us to visually confirm the transition from free-flowing emulsions into semisolid emulgels with high apparent yield stress. TIM–Tgel values showed similar tendencies to these observed through the algorithmic analysis of viscosity–temperature profiles. The actual hard gels were successfully formed in every studied case, although they appeared at temperatures slightly higher than Tgels revealed in previous tests. Nevertheless, no new connections were found between TIM–Tgel values and other rheological measures.

### 3.3. Syringeability

Blank and IBU-loaded ORC emulsions were successfully extruded through every needle at flow rates ranging from 0.25 to 8.0 mL/min. The flow observed at the needle tip was drop-wise, although it was steady and no clogging was observed in any case. For pictures taken during the test, please refer to Figure S9 in Supplementary Materials.

**Figure 11:**
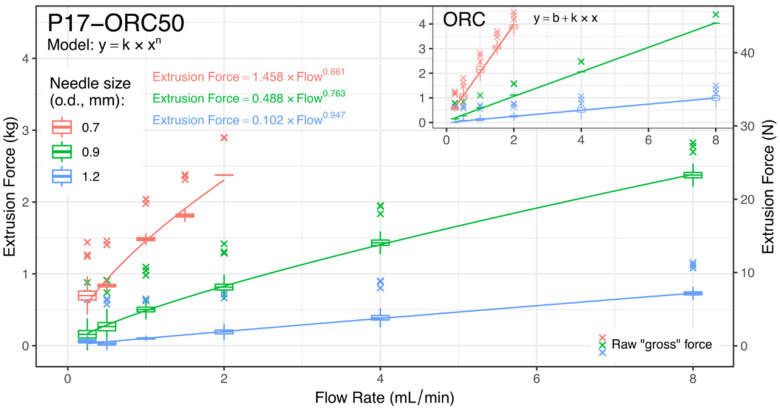
Syringeability study results in P17–ORC50 blank emulsion. Inset shows results obtained for pure ORC oil, with the same y-range as in the main plot. A linear fit is applied for pure ORC. Models and box-and-whiskers plots were calculated upon net extrusion force, while raw (gross) data was also shown for a better consideration of the actual force needed to move the plunger.

The syringeability study performed on a blank P17–ORC50 emulsion confirmed its pseudoplastic behaviour, which was especially visible in conditions of the highest shear rate, i.e., with the thinnest needle (0.7 mm/22 G). A non-linear power model was fitted with a satisfactory result (see Figure 11). The *n* parameter, which could be considered here a (reverse) measure of pseudoplasticity, was significantly lower in IBU-loaded emulsions (compared with Figure S8 in Supplementary Materials), which at the study temperature (25 ± 1 °C) were all present in the gel state. The *n* value seemed also to negatively correspond to the viscosities measured at 25 °C, and not to the Pseudoplasticity Index, although no strong correlation was found. While obtaining a “yield” force should be expected in gelated IBU-loaded emulsions, a model accounting for it ($y=b+k\times x^{n}$) brought no improvement in general data representation and was considerably more susceptible to errors caused by local data deviations.

At the same time, the pure ORC (see inset in Figure 11) and every ORC–IBU solution showed a clearly linear relationship between the flow rate and the force needed to maintain it. Such an observation was expected since the ORC and ORC–IBU solutions were considered Newtonian fluids. A linear model was fitted for each formulation-needle set with excellent results, usually showing no statistically significant intercept. This proved that the method for measurement and subtraction of the “empty syringe” force was correct, even though empty syringes tended to behave in a difficult, non-linear way. However, some general deviations were noticed, for both oily solutions and emulsions, mainly in the low-flow and/or low-force region.

A comparison between emulsions and ORC (or ORC–IBU solutions) reveals a distinct advantage of a two-phase system due to the significant viscosity drop occurring under shear. Every tested emulsion needed significantly lower net force than even pure ORC (Table 5). A remarkable difference was visible with a 0.9 mm (20 G) needle, where the mean forces noted for emulsions were from 1.7 to 2.06 times lower than for their respective ORC/ORC–IBU solution. A one-way ANOVA statistic was calculated upon a part of the data presented in Table 5. To avoid over-comparison, the analysed groups were narrowed to contain ORC-only emulsions and pure ORC. After the general differences were confirmed, the data was further analysed using the Holm’s post-hoc method. The dissimilarities between different emulsions turned out to be mostly insignificant (*p*-values not presented here), but the more interesting difference—which was between emulsions and ORC—was of very high significance in 2/3 of cases. The *p*-values for comparisons of *each emulsion vs. ORC* are presented in Table 5. Unfortunately, relatively wide data distribution in group 1.2/8.0 led to no statistical significance at *p* < 0.05.

### 3.4. Drug Release Kinetics

**Figure 12:**
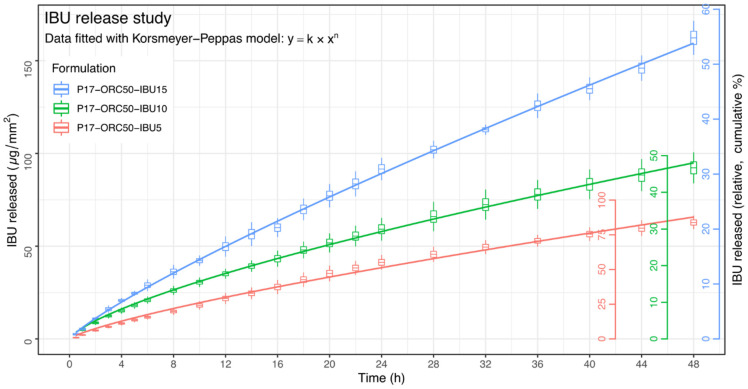
IBU release results. Means from six units of every formulation, ±std. error (boxes), ±95% confidence interval (whiskers). Right-side y-axes refer to the cumulative, relative % of IBU released from each formulation.

Due to a well-defined diffusion area and different levels of IBU in each emulsion, the results of the drug release study were presented in their relatively raw form, i.e., as µg of IBU released per 1 mm^2^ of membrane’s surface area. A brief graphical presentation is shown in Figure 12, and the numerical data is available in Table S2. These methods of data presentation are in accordance with the European Medicines Agency’s (EMA) “Guideline on quality and equivalence of topical products” [52].

A quick comparison between the µg/mm^2^ values and the absolute IBU content in given formulations reveals that the final percent released was 83.88%, 46.68% and 54.79% in P17–ORC50–IBU5, -IBU10 and -IBU15, respectively. Although the “ideal” amount released suggested by EMA (≥70%) was reached only in the case of the lowest strength (P17–ORC50–IBU5), the remaining requirements for an In Vitro Release Test were fulfilled in all formulations, i.e., in every case at least six time points were obtained in the linear portion of the drug release profile [52]. A prolonged release phenomenon was evident in each case. The apparent release rates varied between formulations, yet they were rather steady, and no significant burst occurred in the initial hours; thus, the release kinetics were quite different from those observed usually in pure poloxamer 407 gels [58,59]. Higuchi’s model (“square root of time”, see Table 6 and Table 7) was not applicable, probably due to complicated release mechanics caused by the presence of a dispersed, biphasic drug reservoir.

The plateau of the release profile was not visible in any case. This might be caused by the limited test duration or by the generally anomalous release kinetics, which is discussed later. Due to the risk of a significant back-diffusion of the receptor media and consequent dilution of the hyperosmotic emulgel matrix, the test was not extended beyond 48 h.

To obtain any numerical measure of the release kinetics, six different kinetic models (Table 6) were selected and fitted upon the data in order to choose the one appropriate for further comparisons. The modelling was performed upon the means from six units of every formulation via a non-weighted, non-linear least squares method, and the quality of fit was measured through the Residual Sum of Squares. To perform a proper inter-formulation comparison of the goodness of each fit, the raw data points were standardised to a common range of 0:100 µg/mm^2^, where 100 µg/mm^2^ was assigned to the highest value noted in a given formulation, and all remaining points were adjusted proportionally. The results of this analysis are shown in Table 7. Although the Hixson–Crowell kinetics seemed appropriate for P17–ORC50–IBU5 emulsion, it was evident that the release kinetics in any other case did not strictly follow any of the single-parameter models, and especially not the Higuchi’s diffusion model. Therefore, due to the lowest mean RSS among tested formulations, the Korsmeyer–Peppas model was chosen for an inter-formulation comparison of kinetic parameters performed upon raw data. Model fits were calculated again, and their graphical results are shown in Figure 12 and Table 8.

It is evident that P17–ORC50–IBU10 and -IBU15 emulsions shared a very similar *k* parameter, and the only significant difference between them was in the *n* power resulting in a visible difference in the—however moderate—curvature of their profiles, as may be seen in Figure 12. At the same time, the P17–ORC50–IBU5 emulsion was characterised by an inbetween *n* value, with no statistical significance against others, and a distinctively different *k*, with the latter reasonably corresponding to the lowest y-position of this release profile. 

An important matter in considerations of the shape of these release profiles should be their questionable curvature. A closer look at all three profiles in Figure 12 may reveal that, after an initial curved segment, the large remaining part of each profile takes a quasi-linear character. Thus, another analysis was suggested to evaluate this phenomenon and to assess the possibility of applying a segmented fit of different models as a way to provide more information about the release kinetics.

A segmented regression algorithm was applied, in which the first part of each release profile was assigned to the Korsmeyer–Peppas model, and the remaining to a classic linear model (y=k×x+b). The proposed breakpoint was moved along the dataset, from the 3rd to the 20th time-point, and the regression segments were divided accordingly, with the breakpoint being used concurrently as the last point of the K–P fit and as the first point of the linear fit. The quality of these fits was intended to be evaluated again by RSS values, but because the RSS depends strictly on the absolute range of *y*-values to which it corresponds, a false disproportion between the scores of the lower- and upper-region models was expected. The use of a “weighted” regression, with weights equal to the corresponding mean *y*-values, did not solve this problem, and neither did a regression with an analogous custom loss function because the original aim of these approaches was to change the regression algorithm flow, and not the regression score. The best solution found was a non-weighted least-squares regression, but evaluated through a manually calculated “Adjusted RSS” value. To obtain the Adjusted RSS, the raw residuals were divided by their corresponding *y*-values, then squared and summed, as is usually done:

(2) $$\mathrm{Adjusted}\;\mathrm{RSS}=\sum_{i=1}^{n}\;{\left(\frac{y_{i}-{\hat{y}}_{i}}{y_{i}}\right)}^{2}.$$

**Figure 13:**
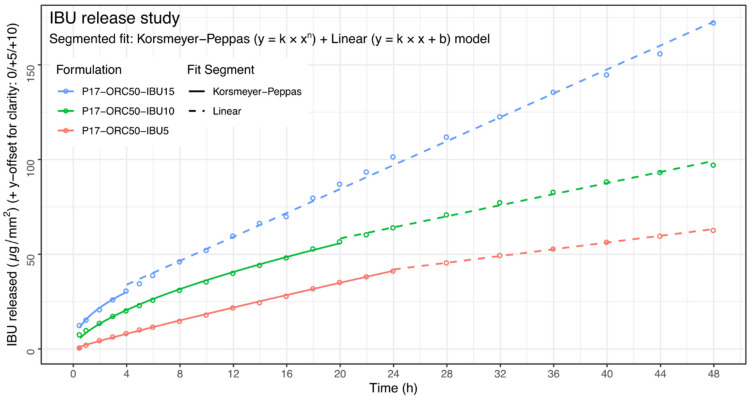
IBU release results with segmented regression lines overlaid. Notice the y-offset applied in different series: +5 and +10 for P17–ORC50–IBU10 and -IBU15, respectively.

Next, the Adjusted RSS values obtained from both models were added at every given time-point, and the lowest sum of Adjusted RSS in each formulation was selected as an indicator of the breakpoint, i.e., the boundary between non-linear and linear fit. The essence of numerical results of these operations is presented in Table 9, while the graphical representation of the final fits is shown in Figure 13. The complete *breakpoint vs. RSS* table is available in Supplementary Materials (Table S3).

The breakpoint values were screened for any connections with other measured qualities of IBU-loaded emulsions. Some significant correlations were found with the viscosities measured at 20 and 25 °C (both negative), as well as with Tgel (positive). However, these do not seem to explain the mechanisms behind the different breakpoint times in any reasonable way.

However, an interesting connection was found inbetween some of the segmented models’ parameters. The *n* obtained from segmented K–P fit was positively, almost perfectly (R^2^ = 0.9999, RSS = 4.4 × 10^−6^) correlated with the k from linear fit; at the same time, both of them turned out to be negatively correlated with the breakpoint time (R^2^ = 0.9981 and 0.9969) and positively with the IBU content (R^2^ = 0.9182 and 0.9246, respectively). Therefore, it appeared that a higher *n* (to be more specific: closer to 1.0), being, in this case, a relative measure of linearity (!) in the non-linear segment, was accompanied by a lower breakpoint time, meaning a quicker onset of the linear region. Ergo, the two linearity measures turned out to be quite convergent, and possibly correlated with the IBU content. 

This hypothesis was tested through linear regression, in which the common measure of linearity was expressed as:

(3) $$\mathrm{Linearity}\;\mathrm{score}=\left(48\;h-\mathrm{breakpoint}\right)\times n_{\;from\;K–P\;segment},$$

and was tested against IBU content. A significant positive correlation was found (*p* = 0.00019, R^2^ = 0.8788). However, due to the limited number of data points, this result should be understood only as an indicator of a general tendency and not as a proof of any causation. Many more results should be collected and included to reveal the actual shape of this correlation, which is probably far from strictly linear.

The drug content undoubtedly positively affected the absolute amount released (µg/mm^2^) in each formulation and the overall linearity score. However, it does not explain the distinct difference in the relative percent released observed between P17–ORC50–IBU5 and the two remaining formulations. After a thorough consideration of the release rates and their corresponding rheological properties, it appears that the faster relative release in P17–ORC50–IBU5 may, in fact, be related to the gelation temperature (Tgel). Because of a strong correlation between the IBU content and Tgel (and also the viscosity at 25 °C; see Figure 7 and Figure 10), the tested formulations were expected to lose a part of their viscosity and gelation capability along with the decrease in IBU content. This drop of viscosity should have eased the diffusion process and therefore accelerated the drug release rate over time, which explains not only the higher relative % released in P17–ORC50–IBU5 but also the over-proportional *k* value in its linear segment (Table 9). A moderate back-diffusion of the receptor medium, which was observed in each sample during the release study, might even exaggerate this effect. A post-release viscosity test could help verifying the above explanation, yet it was impossible to be performed due to a limited amount of emulgel used in the release study. Hence, this curious phenomenon should be studied later in a different setup with larger samples. Also, it must be emphasised that extra caution is necessary with every thermosensitive formulation where the gelation temperature is affected by the API content.

## 4. Conclusions

Poloxamer 407 (Pluronic^®^ F-127) provided an excellent capability to emulsify oily media of different chemical structures. In each case, a 17% (*w*/*w*) aqueous solution of P407 was capable of emulsifying any tested vegetable oil in at least a 1:1 proportion, giving a smooth oil-in-water emulsion and retaining most of its original thermosensitive properties. In addition, every *o*/*w* emulsion was capable of forming a pseudoplastic emulgel, although the gelation temperature and final emulgel strength were slightly affected by the addition of the oil phase. However, the final rheological properties should rather be considered as an outcome of the specific interaction between P407 and a given oil and not of the sole presence of a dispersed phase.

The general rheological behaviour of tested emulsions turned out to be strongly pseudoplastic, with a free-flowing appearance at low temperatures (<20 °C) and a creamy and semisolid texture over their gelation temperature (19–30 °C). Their viscosities dropped 2- to 9-fold over one decade of a shear rate, and this property was even more evident in the gel state. A high degree of pseudoplasticity allowed for a simulated administration through a syringe and needle, with relatively less effort than was necessary for pure castor oil. A flow rate of 8.0 mL/min was achievable in P17–ORC50 and P17–ORC50–IBU5/10/15 emulsions with a 0.9 mm (20 G) needle and a common 2 mL syringe with the gross force not exceeding 3 kg (29.4 N) when expelled into the air.

A 50% castor oil emulsion (P17–ORC50) was capable of incorporating up to 15% (*w*/*w*) of ibuprofen in its internal phase. The drug was successfully released in an in vitro dissolution test in an undoubtedly prolonged manner. Over 48 h, ibuprofen-loaded emulsions provided up to 44 h of controlled, linear drug release preceded by a short period of moderately non-linear behaviour. The release rate and the length of the linear segment were found to be positively dose-dependent, as was the overall release linearity score, which was significantly higher in the more concentrated emulsions.

The composition of oil and poloxamer 407 solution formed a two-phase thermosensitive system capable of incorporating a significant amount of lipophilic drug and releasing it in a controlled, prolonged manner. Moreover, the simultaneous presence of aqueous and oily phases may provide convenient reservoirs for both lipophilic and hydrophilic APIs and excipients, making such emulsion an interesting vehicle ready to be transformed into a complex drug form. It is worth noting that the general idea of thermosensitive, poloxamer-based emulgels as drug preparations is not limited to lipophilic APIs dissolved in refined oils but is also open for an incorporation of natural oily extracts and oleoresins of specific pharmaceutical activity, as shown by Campanholi et al. in their recent paper [37].

The convenient physicochemical compatibility of poloxamer 407 and castor oil, with the latter being already an interesting solvent in pharmaceutical technology [60], may be a promising factor for the future development of novel drug products. Oils other than those described here could be emulsified or other excipients introduced to adjust the emulsion’s properties up to the specific needs in the matter of rheological properties and release kinetics. The high-oil-content, thermosensitive emulgel may serve as a potent, controlled-release vehicle for the topical, ocular or subcutaneous administration of lipophilic APIs.

## Figures and Tables

**Table 2:** Detailed composition of formulations. Quantities per 10.0 g of the sample weight.

Formulation	Poloxamer 407 (g)	Water (g)	Oil Type	Oil Content (g)	IBU Content (g)	Initial IBU Content in Oil Phase (%)
P17–PAR50	0.85	4.15	Paraffin	5.00	-	-
P17–OAR50	0.85	4.15	Peanut Oil	5.00	-	-
P17–ORP50	0.85	4.15	Canola Oil	5.00	-	-
P17–ORC50–IBU5	0.85	4.15	Castor Oil	4.75	0.25	5.0
P17–ORC50–IBU10	0.85	4.15	Castor Oil	4.50	0.50	10.0
P17–ORC50–IBU15	0.85	4.15	Castor Oil	4.25	0.75	15.0

**Table 3:** Summary of rheological data. Means ± sd, *n* = 3. Viscosities (v) in mPas; Pseudoplasticity Index (PI)—dimensionless.

Formulation	v20 °C 38.4 s^−1^	v25 °C 38.4 s^−1^	v25 °C 0.384 s^−1^	v37 °C 0.384 s^−1^	PI 20 °C	PI 25 °C	PI 37 °C	Tgel	TIM-Tgel
P17	48.8 ± 1.3	1560 ± 30	-	359,000 ± 6000	0.98 ± 0.009	6 ± 0.3	8.9 ± 0.14	25.17 ± 0.04	25.5
P17–PAR50	886 ± 15	1641 ± 8	-	222,000 ± 5000	3.035 ± 0.014	1.879 ± 0.013	7.76 ± 0.06	29.4 ± 0.7	30.5
P17–OAR50	595 ± 4	1022 ± 10	-	188,800 ± 1100	3.067 ± 0.013	1.953 ± 0.006	7.38 ± 0.04	27.9 ± 0.2	29.5
P17–ORP50	679 ± 3	1061 ± 3	-	215,000 ± 4000	3.061 ± 0.005	1.794 ± 0.003	7.57 ± 0.08	29.5 ± 0.3	30.5
P17–ORC50	720 ± 18	2100 ± 90	-	367,000 ± 6000	3.23 ± 0.03	1.923 ± 0.012	8.888 ± 0.006	25.83 ± 0.06	26.5
P17–ORC50–IBU5	990 ± 20	-	256,000 ± 10,000	426,700 ± 900	1.941 ± 0.007	8.29 ± 0.05	9.01 ± 0.06	23.5 ± 0.2	24.0
P17–ORC50–IBU10	1194 ± 11	-	291,000 ± 4000	430,000 ± 11,000	3.052 ± 0.012	8.61 ± 0.14	8.803 ± 0.019	21.51 ± 0.03	21.5
P17–ORC50–IBU15	2210 ± 20	-	358,000 ± 4000	426,000 ± 2000	5.94 ± 0.03	7.99 ± 0.02	8.818 ± 0.002	18.98 ± 0.09	20.5

**Table 4:** Summary of viscosities measured in ORC and ORC–IBU solutions. Means ± sd, *n* = 4, 8, 18 (respectively at 20, 25 and 37 °C).

Formulation	v20 °C (3.75–22.5 s^−1^)	v25 °C (3.75–33.75 s^−1^)	v37 °C (3.75–82.5 s^−1^)
ORC	1044 ± 8	705 ± 5	299.9 ± 1.7
ORC–IBU5	1034 ± 8	707 ± 3	296 ± 2
ORC–IBU10	1054.7 ± 1.4	717 ± 4	306 ± 2
ORC–IBU15	1072 ± 5	712 ± 4	302 ± 4

**Table 5:** Summary of syringeability study. Net Extrusion Forces, kg, shown as means ± sd, *n* = 3. Only the highest flow rates are shown for every needle size. Brackets contain the *p*-values obtained from post-hoc comparisons between emusions and pure ORC (in respective columns/rows). General ANOVA *p*-values from group comparisons were 1.7 × 10^−7^, 2.1 × 10^−9^ and 4 × 10^−2^ for 0.7, 0.9 and 1.2 mm needle, respectively.

Formulation	Needle Size (mm) (Gauge)/Flow (mL/min)		
	0.7 (22 G)/2.0	0.9 (20 G)/8.0	1.2 (18 G)/8.0
P17–ORC50	2.375 ± 0.003	2.37 ± 0.06	0.73 ± 0.04
	(2.4 × 10^−7^)	(4.7 × 10^−9^)	(0.07)
P17–ORC50–IBU5	2.55 ± 0.08	2.45 ± 0.09	0.75 ± 0.08
	(5.1 × 10^−7^)	(5.8 × 10^−9^)	(0.09)
P17–ORC50–IBU10	2.78 ± 0.08	2.56 ± 0.06	0.88 ± 0.07
	(1.8 × 10^−6^)	(8.2 × 10^−9^)	(0.44)
ORC	3.89 ± 0.14	4.03 ± 0.016	0.99 ± 0.16
ORC–IBU5	3.763 ± 0.019	4.19 ± 0.05	0.94 ± 0.03
ORC–IBU10	4.54 ± 0.16	5.27 ± 0.11	1.024 ± 0.002

**Table 6:** Equations of applied kinetic models.

Model Name + Abbreviation, If Used	Model Equation	Transformed Equation Used in Estimations
Zero-order kinetics	Q = *k* × t	% (or µg) released = *k* × t
First-order kinetics	Q = Q_0_ × e^−*k*t^	% released = 100 − 100 × e^−*k*t^
Higuchi’s model	Q = a × t^1/2^	% (or µg) released = a × t^1/2^
Hixson-Crowell model	Q^1/3^ = Q_0_^1/3^ − *k* × t	% released = 100 − (100^1/3^ − *k* × t)^3^
Korsmeyer–Peppas model, K–P	Q = *k* × t*^n^*	% (or µg) released = *k* × t*^n^*
Simplified K–P model, t^2/3^	Q = *k* × t^2/3^	% (or µg) released = *k* × t^2/3^

**Table 7:** Release kinetics, model fit summary. Modelling was performed upon the averaged data from six units of every formulation. Released amounts were normalised to a range of 0:100 for each formulation to provide a unified RSS result allowing for inter-formulation comparison of model fit.

Formulation	RSS (Residual Sum of Squares) in Each Model					
	Zero Order Kinetics	First Order Kinetics	Higuchi	Hixson–Crowell	Korsmeyer–Peppas	Simplified K–P Model
P17–ORC50–IBU5	745.0	282.8	1549.6	46.7	117.1	305.4
P17–ORC50–IBU10	944.3	81.4	826.4	50.5	19.7	52.2
P17–ORC50–IBU15	258.1	419.5	1659.2	162.3	13.8	376.4

**Table 8:** Statistical summary of Korsmeyer–Peppas model fit. Raw (µg/mm^2^) data used; means ± sd, from six units of each. *p*-values from pairwise comparisons in ANOVA post-hoc test (Holm’s method).

Formulation	*k* Estimate	P vs. IBU10	P vs. IBU15	*n* Estimate	P vs. IBU10	P vs. IBU15
P17–ORC50–IBU5	3.3 ± 0.9	0.0012	0.0010	0.78 ± 0.06	0.0988	0.0988
P17–ORC50–IBU10	6.1 ± 1.1	-	0.7742	0.71 ± 0.05	-	0.0024
P17–ORC50–IBU15	6.3 ± 1.3	0.7742	-	0.84 ± 0.05	0.0024	-

**Table 9:** A summary of segmented fit results.

Formulation	Breakpoint Time (h)	Adjusted RSS			Model Equations	
		K–P	Linear	Sum	K–P	Linear
P17–ORC50–IBU5	24	0.53190	0.00047	0.5324	y = 6.18 × x^0.60^	y = 0.90 × x + 20.6
P17–ORC50–IBU10	20	0.04065	0.00257	0.0432	y = 7.34 × x^0.66^	y = 1.46 × x + 24.1
P17–ORC50–IBU15	4	0.01308	0.04428	0.0574	y = 6.23 × x^0.84^	y = 3.16 × x + 11.3

## Data Availability

Not applicable.

## Supplementary Materials

The following are available online at www.mdpi.com/article/10.3390/ma14237266/s1, Figure S1. IBU solubility in tested media (mg/g). For abbreviations, see Table 1 in the paper. Means, ±std. error, ±95% confidence interval; *n* = 3; Figure S2. Pseudoplasticity indices at 20 °C. Means, ±std. error, ±95% confidence interval; *n* = 3; Figure S3. Pseudoplasticity indices at 25 °C. Means, ±std. error, ±95% confidence interval; *n* = 3; Figure S4. Pseudoplasticity indices at 37 °C. Means, ±std. error, ±95% confidence interval; *n* = 3; Figure S5. Mixtures of P17 and chosen natural oils, pre- and post-emulsification. Photographed in identical light conditions; Figure S6. Mixtures of P17 and chosen semisynthetic oils, pre- and post-emulsification. Photographed in identical light conditions; Figure S7. P17–ORC50 emulsion compared with IBU–loaded ORC emulsions; Figure S8. Syringeability study results in P17–ORC50–IBU10 emulsion. Models and box-and-whiskers plots were calculated upon net extrusion force, while raw (gross) data was also shown for a better consideration of the actual force needed to move the plunger. Means, ±std. error, ±95% confidence interval; *n* = 3; Figure S9. Extrusion process observed at the needle-end in syringeability study: (A) drop-wise flow in a blank emulsion; (B) formation and detachment of elongated drops in a gelated, IBU-loaded emulsion. Needle: 20 G (0.9 mm × 40 mm); flow rate: 0.5 mL/min; temperature: 25 °C; Table S1. Specification of needles used in the syringeability study; Table S2. Complete results of the drug release study. IBU released, µg/mm^2^. Means ± sd, *n* = 6; Table S3. Complete results of segmented-fit modelling of the drug release kinetics; summary of adjusted-RSS values against proposed breakpoints. Values in bold are the minima found among the sums of Adjusted-RSS from non-linear and linear fits. K–P = Korsmeyer–Peppas model: *y* = *k* × *x^n^*; Linear: *y* = *k* × *x*.
