# Supplementary material for: Poloxamer 407-Based Thermosensitive Emulgel as a Novel Formulation Providing a Controlled Release of Oil-Soluble Pharmaceuticals—Ibuprofen Case Study
Source: Materials (Basel). 2021 Nov 27;14(23):7266. doi: 10.3390/ma14237266 (PMC8658182; doi:10.3390/ma14237266)
Supplement: Supplementary file 1 [file materials-14-07266-s001.zip › materials-1434629-supplementary.pdf]

materials

Poloxamer 407-Based Thermosensitive Emulgel as a Novel Formulation Providing a Controlled Release of Oil-Soluble Pharmaceuticals—Ibuprofen Case Study

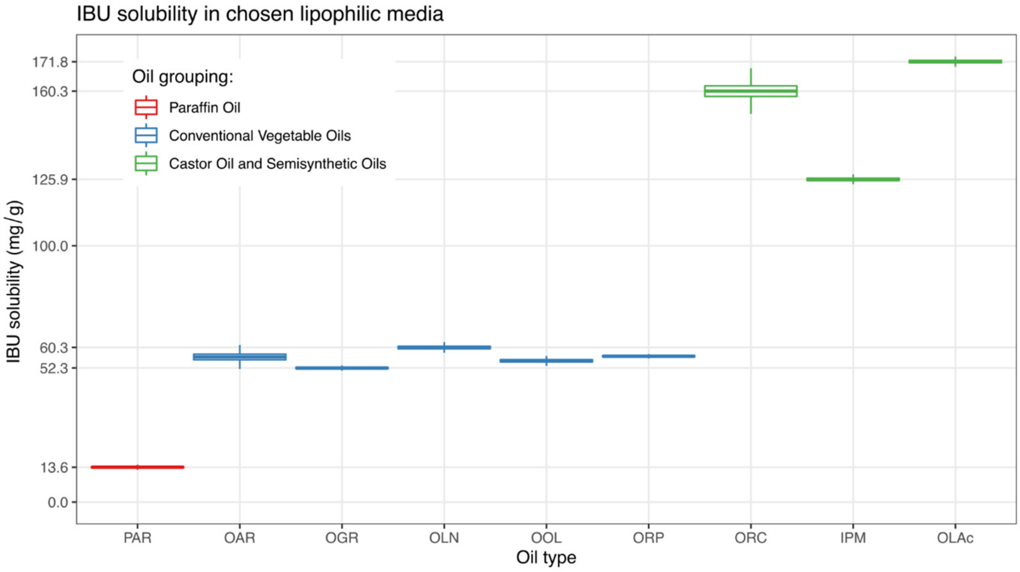

Figure S1. IBU solubility in tested media (mg/g). For abbreviations, see Table 1 in the paper. Means, ±std. error, ±95% confidence interval; *n* = 3.

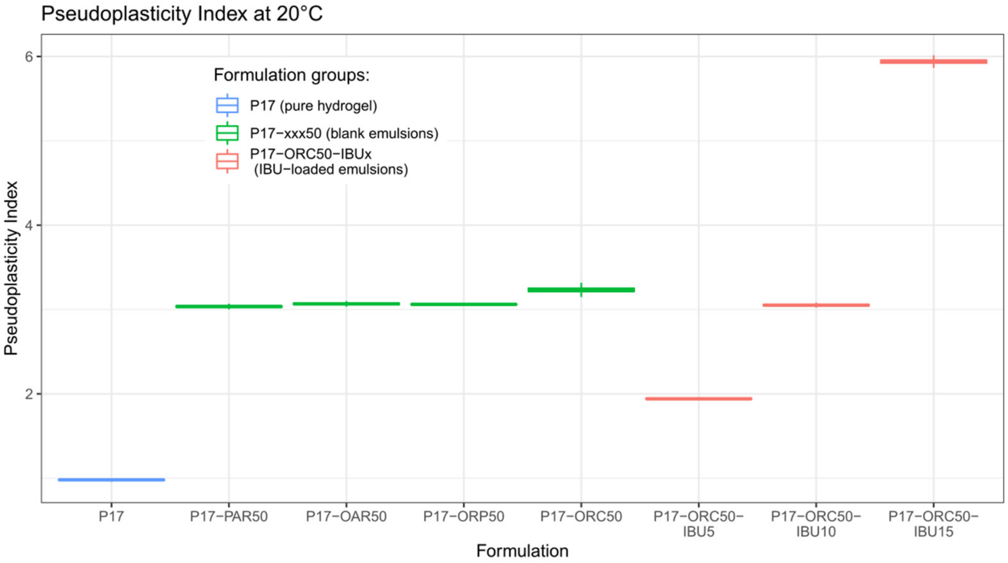

Figure S2. Pseudoplasticity indices at 20 °C. Means, ±std. error, ±95% confidence interval; *n* = 3.

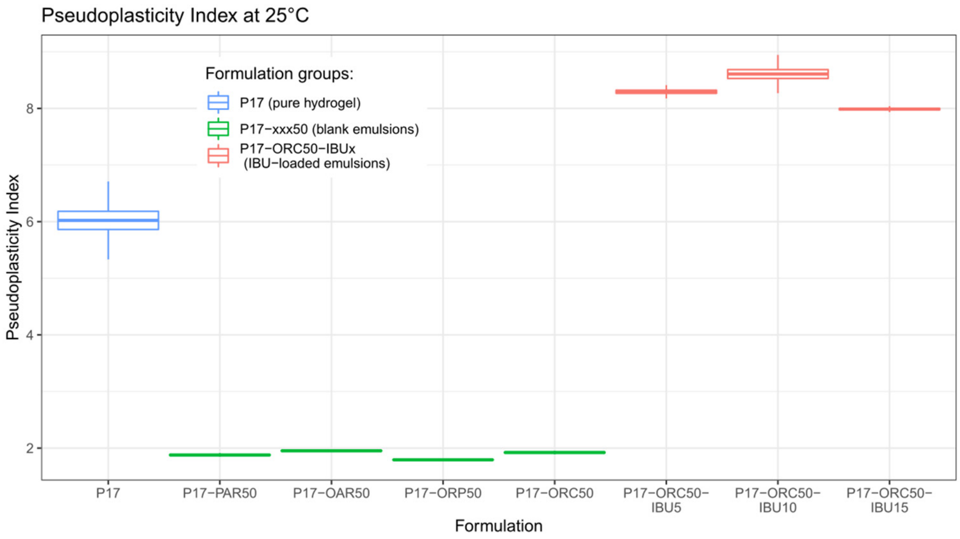

Figure S3. Pseudoplasticity indices at 25 °C. Means, ±std. error, ±95% confidence interval; *n* = 3.

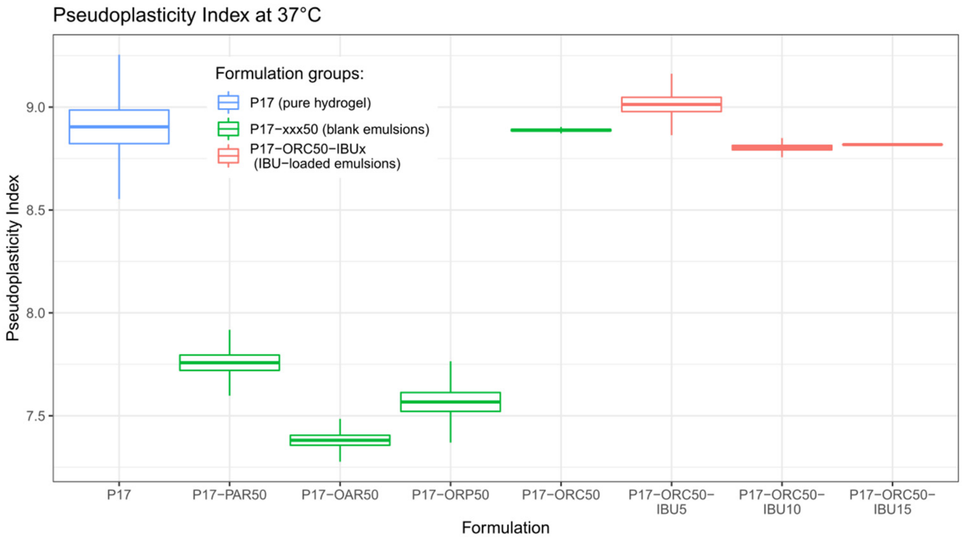

Figure S4. Pseudoplasticity indices at 37 °C. *Means*, ±std. error, ±95% confidence interval; *n* = 3.

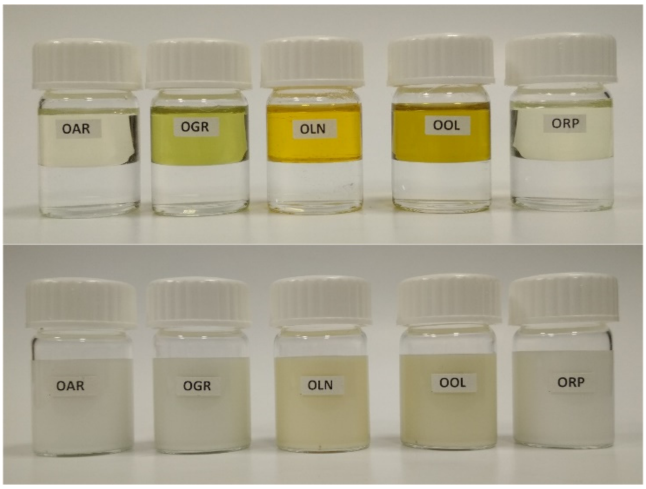

Figure S5. Mixtures of P17 and chosen natural oils, pre- and post-emulsification. Photographed in identical light conditions.

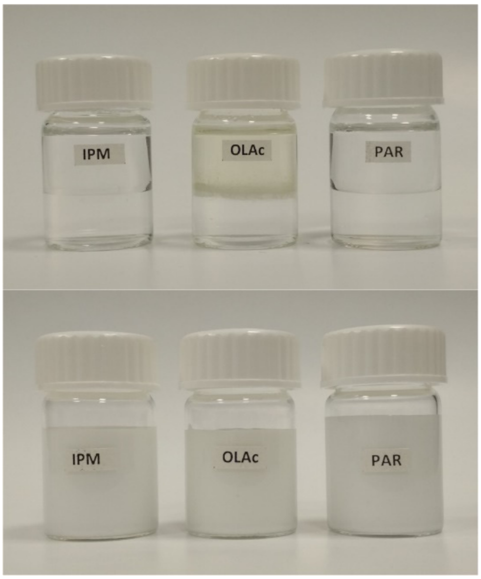

Figure S6. Mixtures of P17 and chosen semisynthetic oils, pre- and post-emulsification. Photographed in identical light conditions.

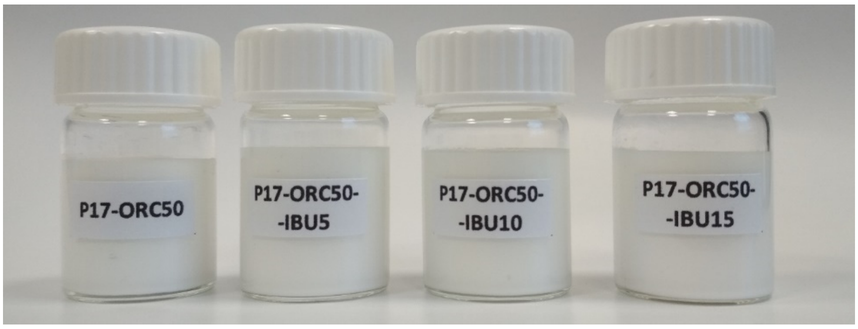

Figure S7. P17–ORC50 emulsion compared with IBU–loaded ORC emulsions.

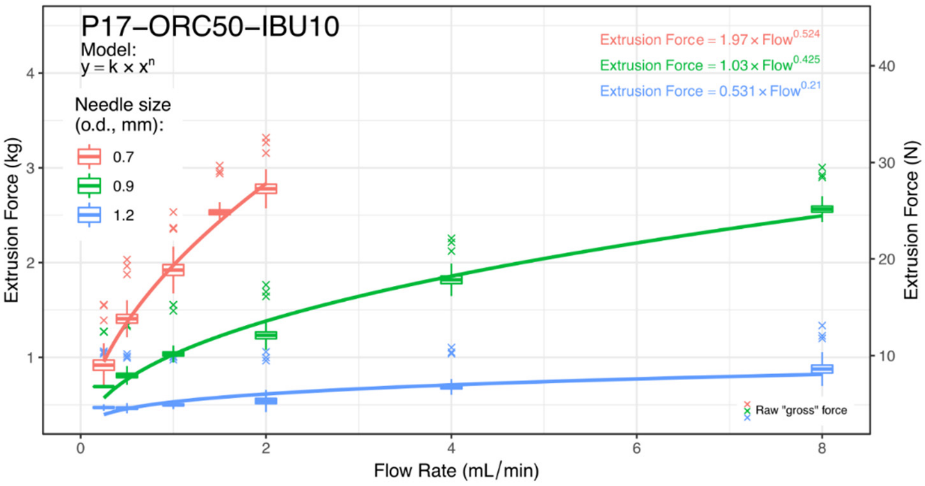

Figure S8. Syringeability study results in P17–ORC50–IBU10 emulsion. Models and box-and-whiskers plots were calculated upon net extrusion force, while raw (gross) data was also shown for a better consideration of the actual force needed to move the

plunger.Means, ±std. error, ±95% confidence interval; *n* = 3..

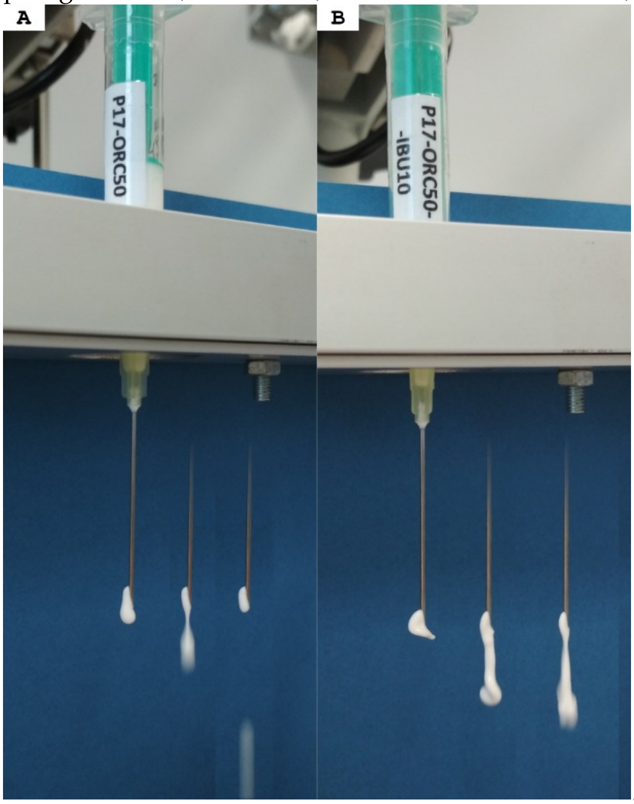

.

Figure S9. Extrusion process observed at the needle-end in syringeability study: (**A**) drop-wise flow in a blank emulsion; (**B**) formation and detachment of elongated drops in a gelated, IBU-loaded emulsion. Needle: 20 G (0.9 mm x 40 mm); flow rate: 0.5 mL/min; temperature: 25 °C.

Table S1. Specification of needles used in the syringeability study.

Needle Gauge	Outer Diameter (mm)	Inner Diameter (mm)	Declared Length (mm)	Actual (measured) Capillary Length (mm)
18 G	0.7	0.413	30	36.0
20 G	0.9	0.603	40	42.0
22 G	1.2	0.838	40	41.5

Table S2. Complete results of the drug release study. IBU released, μg/mm². Means ±sd, *n* = 6.

Time (h)	IBU Released (μg/mm²)		
	P17–ORC50–IBU5	P17–ORC50–IBU10	P17–ORC50–IBU15
0.5	0.7 ± 0.5	2.6 ± 0.4	2.5 ± 1.1
1	2.1 ± 0.5	4.9 ± 0.8	5 ± 1
2	4.6 ± 0.7	8.7 ± 1.2	10.9 ± 0.9
3	6.5 ± 0.8	12.4 ± 1.3	16.1 ± 1.9
4	8.3 ± 1	15.3 ± 1.6	20.7 ± 1.3
5	10.2 ± 1.2	18.1 ± 1.7	24.6 ± 1.3
6	11.7 ± 1.2	20.9 ± 1.9	29 ± 3
8	14.8 ± 1.7	26 ± 2	36 ± 4
10	18 ± 2	31 ± 2	42 ± 3
12	22 ± 3	35 ± 3	50 ± 5
14	25 ± 3	39 ± 3	57 ± 6
16	28 ± 4	43 ± 4	60 ± 5

18	32 ± 4	48 ± 4	70 ± 6
20	35 ± 4	52 ± 5	77 ± 6
22	38 ± 4	55 ± 5	84 ± 6
24	41 ± 4	59 ± 6	92 ± 6
28	46 ± 4	66 ± 8	102 ± 4
32	50 ± 4	72 ± 8	113 ± 3
36	53 ± 3	78 ± 7	126 ± 6
40	57 ± 4	83 ± 8	135 ± 6
44	60 ± 4	88 ± 8	146 ± 6
48	63 ± 4	92 ± 8	162 ± 9

Table S3. Complete results of segmented-fit modelling of the drug release kinetics; summary of adjusted-RSS values against proposed breakpoints. Values in bold are the minima found among the sums of Adjusted-RSS from non-linear and linear fits. K–P = Korsmeyer–Peppas model: $y = k \times x^n$; Linear: $y = k \times x$.

Break-point (h)	P17–ORC50–IBU5			P17–ORC50–IBU0			P17–ORC50–IBU15		
	K–P	Linear	Sum	K–P	Linear	Sum	K–P	Linear	Sum
2	0.0657	9.52e-01	1.0176	0.000247	0.738791	0.7390	0.00112	0.296763	0.2979
3	0.2020	5.13e-01	0.7147	0.000165	0.325924	0.3261	0.00407	0.094336	0.0984
4	0.3439	3.41e-01	0.6846	0.002249	0.199445	0.2017	0.01308	0.044287	**0.0574**
5	0.4368	2.47e-01	0.6839	0.005930	0.126883	0.1328	0.03998	0.027685	0.0677
6	0.6033	1.92e-01	0.7958	0.008357	0.077987	0.0863	0.05690	0.016596	0.0735
8	0.8629	1.28e-01	0.9912	0.012246	0.040505	0.0528	0.11378	0.011955	0.1257
10	0.9297	8.21e-02	1.0118	0.022789	0.025806	0.0486	0.20935	0.010584	0.2199
12	0.7930	5.08e-02	0.8438	0.029364	0.016292	0.0457	0.22640	0.008160	0.2346
14	0.8189	3.64e-02	0.8553	0.037106	0.011039	0.0481	0.23549	0.007691	0.2432
16	0.7780	2.04e-02	0.7983	0.045095	0.007122	0.0522	0.34877	0.007550	0.3563
18	0.6399	8.54e-03	0.6484	0.040613	0.003724	0.0443	0.28489	0.001996	0.2869
20	0.5562	3.85e-03	0.5601	0.040655	0.002576	**0.0432**	0.22457	0.001239	0.2258
22	0.5325	1.66e-03	0.5342	0.042831	0.001746	0.0446	0.19055	0.001020	0.1916
24	0.5319	4.70e-04	**0.5324**	0.045188	0.001075	0.0463	0.14592	0.000849	0.1468
28	0.7109	1.08e-04	0.7110	0.053486	0.000431	0.0539	0.14780	0.000698	0.1485
32	1.0736	3.11e-05	1.0736	0.066624	0.000139	0.0668	0.17073	0.000641	0.1714
36	1.6247	2.46e-05	1.6248	0.093163	0.000080	0.0932	0.16813	0.000644	0.1688
40	2.2452	1.09e-06	2.2452	0.126833	0.000025	0.1269	0.19789	0.000213	0.1981
